# Dual processes, dual virtues

**DOI:** 10.1007/s11098-021-01761-7

**Published:** 2021-12-01

**Authors:** Jakob Ohlhorst

**Affiliations:** 1 University of Cologne, Cologne, Germany; 2 University of Glasgow, Glasgow, UK

**Keywords:** Virtue epistemology, Dual-process theory, Reliabilism, Responsibilism, Cognitive psychology

## Abstract

I argue that virtue reliabilism and virtue responsibilism are complementary. They do not give competing accounts of epistemic virtue. Rather they explain the excellent functioning of different parts of our cognitive apparatus. Reliabilist virtue designates the excellent functioning of fast and context-specific Type 1 cognitive processes, while responsibilist virtue means an excellent functioning of effortful and reflective Type 2 cognitive processes. This account unifies reliabilist and responsibilist virtue theory. But the virtues are not unified by designating some epistemic norm that both aim at. Instead, I unify them through their cognitive foundations. Because Type 1 and Type 2 cognition are complementary, reliabilist and responsibilist virtues are complementary. Thereby, this dual-process theory of epistemic virtue gives a naturalised account of virtues as well as an explanation of how reliabilism and responsibilism relate. This approach offers a solution for both the generality problem and the situationist challenge to virtue epistemology; additionally it preserves the epistemological autonomy of each virtue type.

## Introduction

Virtue epistemology is split down the middle. On the one hand, we have virtue reliabilism, arguing that epistemic virtues are faculties that reliably deliver the truth. On the other hand, there is virtue responsibilism, arguing that virtues are habits which guarantee that we are intellectually responsible. These two things seem to be of entirely different sorts, and often they are taken to be incompatible.

In this paper, I argue that, despite the appearance of incompatibility, reliabilist virtues and responsibilist virtues are *complementary*. In order to see this, we need to look at the foundations of the two kinds of virtue. These foundations explain how reliabilism and responsibilism do not compete as accounts even though they appear to be incompatible.

The foundations of our epistemic virtues are the cognitive processes that produce our doxastic states. According to *dual-process theories* in cognitive psychology, there are two types of cognitive processes: the fast, automatic, and context-specific processes of *System 1* and the slow, effortful, and reflective processes of *System 2* (Evans & Stanovich, 2013; Kahneman, 2011). For all we know, these are not actually two distinct cognitive or neural systems; rather they are two ways of functioning, two process *types.* I shall argue that the reliabilist virtues belong to processes of *Type 1* while responsibilist virtues belong to processes of *Type* 2.

This proposal unifies and naturalises the two virtue accounts by pointing to their place within an organism’s cognitive make-up. The epistemic virtues are complementary because the dual process types are complementary. The appearance of incompatibility of the virtue accounts arises out of the different nature of the two process types.

As will become clear, this account is somewhat revisionary about what epistemic virtues are exactly, changing the extension of both what counts as a reliabilist and what counts as a responsibilist virtue. My approach also differs from earlier attempts at integrating the two virtue types which focused on the normative profile of the virtues (Fleisher, 2017; Lepock, 2011; Mi & Ryan, 2020; Sosa, 2015). Instead of looking at the normative profile of the virtues, reliabilist and responsibilist virtues are distinguished by their different cognitive foundations.

At the same time, the project is conservative in the sense that it follows and preserves the intuitions and arguments that led to the creation of these two incompatible accounts of virtue at the outset (cf. Axtell, 2017, p. 11). I do not aim to convince either the hardcore reliabilist or the hardcore responsibilist who reject the other view entirely. Instead, my goal is to propose how reliabilist and responsibilist virtue can be brought to work together in a unified naturalised system (cf. Turri et al., 2019).

## Split virtues

Already in its early years, virtue epistemology split. Only seven years after Ernest Sosa’s foundational ‘The Raft and the Pyramid’ (1980) which started the programme of (reliabilist) virtue epistemology, Code (1987) and Montmarquet (1987) published the first formulations of virtue responsibilism.

Traditionally, reliabilist virtues are conceived of as faculties that reliably deliver the truth. Take for example the faculty of vision that reliably delivers information about the shape and colour of objects surrounding us. Reliability here means that the faculty delivers more true than false beliefs. Reliable faculties are virtues because their deliverances’ success can be credited to the person possessing the faculty. Meanwhile, in this traditional account, the epistemic agent does not play a role.

In recent years, reliabilism has developed into a more agential direction (Greco, 2010). Sosa (2015) considers virtues to be *competences* to reliably succeed at something and grants responsibilist character virtues a place of honour as *auxiliary* traits. This view is called agent reliabilism.

Responsibilist virtues in contrast are taken to be habits or traits that are grounded in motivation: a love of truth or of knowledge. They more closely resemble the Aristotelian moral virtues (Aristotle, 2004), and they are a result of the project of introducing the lessons drawn from virtue ethics into virtue epistemology (Zagzebski, 1996). Classic examples of responsibilist virtues are conscientiousness or intellectual humility.

Given that they are habits, we have to acquire, train, and cultivate our responsibilist virtues. Whether they need to be reliable is more contested. Some authors argue that virtue has an epistemic function independent of reliability (Baehr, 2011, p. 12; Battaly, 2015; Montmarquet, 1987; Wright, 2010). If a belief is formed through the exercise of a responsibilist virtue, then the subject has been responsible in forming this belief. The subject therefore is praiseworthy even if the belief was not formed by a reliable process.

Additionally, responsibilist virtues do not account for our innate faculties like vision. We cannot cultivate a habit of seeing. At best we might cultivate a habit of looking closely, but this habit will rely on the reliabilist faculty of seeing. Consequently, responsibilist virtues do not cover all of our cognitive capacities.

This brief account already shows how disparate reliabilist and responsibilist virtues are. The former are faculties or competences which can involve sensory organs; some of these reliable faculties are pure products of evolution, and their norm is whether they *reliably deliver the truth*.^1^ The latter are habits—things we tend to do. They are learnt and habituated, and their principal norm is a *desire for truth* rather than reliable truth delivery itself.

While they are fundamentally different, both appear to get some things right about epistemic virtue. Both theories describe epistemically valuable traits or capacities of epistemic agents, but each separately seems to be lacking in some aspects. We can fruitfully compare this to Alston’s (1993, 2005) pluralist approach to justification.^2^

But, how would we even begin accounting for these disparate things in a unified way? The reliabilist virtues are grounded in reliable concrete faculties or competences that we possess. Responsibilist virtues seem more grounded in what we are abstractly motivated and habituated to do. They require a certain *motivation* while reliabilist virtues may function without even awareness.

For these reasons, the two accounts are usually taken to be incompatible. Some argue for example that the two projects aim for completely disparate things, a position that Baehr (2011) calls ‘autonomous virtue epistemology’. But, there is a trend towards more ecumenical accounts of reliabilism and responsibilism (Axtell, 1997; Battaly, 2008, 2015; Greco, 2010; Lepock, 2011; Sosa, 2015).

However, I will argue that this ecumenism currently lacks a strong enough theoretical foundation. The current ecumenical accounts’ weakness lies in the fact that they attempt to unify the epistemic virtues by arguing that, ultimately, both reliabilism and responsibilism serve to satisfy one single normative goal. Thus, such a teleological ecumenism posits some normative values or goals that both reliabilist and responsibilist virtues must satisfy, albeit in different ways. Goldman (2002) calls such approaches to virtue *moderate thematic unitarianisms*.

I believe that this teleological project is inherently unstable because either the unificatory goal that both virtue types contribute to is the primary goal of one of the virtues, and the other virtue is annexed to the former which effectively eliminates one category of the virtues; or the different virtue types’ goals have nothing in common, and the account simply postulates that all of these values contribute to epistemic virtue. I will illustrate these two instabilities with Lepock’s (2011) unification of the epistemic virtues and Battaly’s (2015) pluralism. My proposal on the other hand is foundational instead of teleological: it brings reliabilist and responsibilist virtues together through their foundation, i.e. our cognitive capacities.

I know of two accounts that explicitly aim at *unifying* the epistemic virtues (Lepock, 2011; Mi & Ryan, 2020). Christopher Lepock (2011) proposes that epistemic virtue is unified by aiming at *significant true belief*, rather than bare reliability. From the norm of significant true belief we can derive both responsibilist and reliabilist virtues. In Goldman’s (2002) terminology, this view is a moderate significant true belief unitarianism.

Lepock distinguishes between low-level virtues and high-level virtues. The former directly and reliably generate true beliefs; for example, my capacity to use *modus ponens* is a reliably truth-preserving low-level virtue. The latter high-level virtues guide and steer the formation of these beliefs. A trait is a virtue if and only if it contributes to an agent’s acquiring significant true beliefs. An agent’s higher-level virtues indirectly contribute to this end by controlling and guiding the lower-level ones to be more reliable and to seek out significant truths. To stay with our example, if I applied modus ponens to every implication-premise pair that came to my mind, I would waste a lot of time and energy on insignificant beliefs. I could form endless chains of useless disjunctions, or infer a great amount of propositions that my tallest friend F is taller than my other friends f_1_, f_2_, f_3_, … f_n_. Consequently, I need to exhibit the high-level virtue of judiciousness when I use *modus ponens* in order to make significant inferences.

This is a form of agent reliabilism. Higher-level virtues are not characterised by the motivation behind them or how an agent possessing them manifests responsibility. Instead, they extensionally coincide with the virtues that are usually considered to be responsibilist. Consequently, Lepock’s analysis sacrifices responsibilism’s key characteristics for a significant true belief-reliabilism. It is less of a unification than an annexation of responsibilism to reliabilism. As I will argue further below, Mi’s and Ryan’s (2020) account does the same but under responsibilist auspices.

A similar issue arises for Sosa’s (2015) and Fleisher’s (2017) agent reliabilism which restricts *epistemology* to reliabilist virtues while auxiliary responsibilist virtues play a heuristic role. They are methodological tools aiding discovery. Responsibilist virtues are mere handmaidens to the reliabilist competences.

Battaly (2015) defends a pluralist account of virtue. Virtues are unified insofar as they are excellences of a person. Battaly distinguishes two ways how a trait may be an excellence: it can be a virtue because it leads to good ends or it may be a virtue because it involves good motivations. This traces the distinction between reliabilism and responsibilism. Battaly argues that we need both to fully account for our virtues.

I agree. However, unifying virtues under the heading of *excellences of a person* does not work. Pluralism simply states that there are two disparate kinds of excellence that an agent may possess. For a unification to succeed, the pluralist would have to present an argument why the proposed traits are indeed both epistemic excellences. The problem at hand is well known in debates about pluralism. It is called the “unity challenge” to pluralism (Pedersen, 2017, p. 72).

Thus, teleological ecumenical projects have significant shortcomings. Lepock essentially pitches the reliabilist tent so wide as to extensionally include responsibilist virtues without accounting for their essential characteristics. Battaly unifies virtues under the heading of excellences without explaining what makes these different things into excellences or virtues.

Cognition as conceived by reliabilism and responsibilism respectively seems very different: in one case it is an organism’s cognitive output, in the other it is taken to be an agent’s achievements. Given that these two types of virtue are very different, I believe that we need a more solid basis to unify them.

Namely, we do not need some common normative goal but a different locus of unification for our epistemic virtues. Using some epistemic norm, for example significant true belief, does not do because the reliabilist and responsibilist epistemic norms are too disparate to be gathered under the tent of a single epistemic norm. There is no single epistemic norm fit to do that job because it would always collapse responsibilist virtue into agent reliabilism or vice versa. Insofar, pluralist ecumenism is correct. For these reasons, I suggest to unify the virtues by focusing on their cognitive foundations instead. This means that we need an account of the epistemic agent whose cognitive capacities incorporate both types of virtue.

## The dual cogniser

What are the cognitive foundations for our epistemic virtues? By cognitive foundations, I mean the substrate in which our epistemic virtues are anchored, for example the foundation of a diamond’s hardness is its molecular structure. Sosa (2015, p. 27) calls this a virtue’s “seat”—in the case of epistemic virtues, it is our cognitive apparatus. They are the processes and structures through which our mind processes information and forms beliefs. In this paper, I will focus on one particular theory of how cognitive psychology models our cognition: dual-process theory. It is one of the most popular accounts available although it is not uncontested. The account I will defend here simply assumes dual-process theory to be true; thus, it entirely depends on it (Evans & Stanovich, 2013; Kahneman, 2011; Stanovich & West, 2000).

Dual-process theory argues that humans process information in two distinct ways. Type 1 information processing is fast, automatic, and context-specific. Type 2 processes are slow, controlled, and rule-guided (Stanovich & West, 2000, p. 658). In folk psychological terms, Type 1 processes would be called intuitive while Type 2 cognition would be considered to be reasoning (Kahneman & Frederick, 2002, p. 51).

The two types are so different that dual-process theorists initially thought that they were subserved by different and distinct systems. They are therefore sometimes called System 1 and System 2. By now, however, dual-process theorists argue instead that the two types describe different modes of processing information (Evans & Stanovich, 2013, pp. 224–225). Table 1 contains some of the properties often ascribed to the two systems.

**Table 1:** Characteristics of systems 1 and 2 (Stanovich & West, 2000, p. 659)

System 1	System 2
Associative	Rule-based
Holistic	Analytic
Automatic	Controlled
Relatively undemanding of cognitive capacity	Demanding of cognitive capacity
Relatively fast	Relatively slow
Acquisition by biology, exposure, and personal experience	Acquisition by cultural and formal tuition

I described Type 1 as fast, automatic, and context-specific. A paradigmatic example for a Type 1 process is our face recognition (Osman, 2004, p. 992). If you see a face, you will automatically form a set of beliefs: you may recognise the person, and you will read their facial expression. All of this happens automatically and immediately. You do not expend any effort or guide the process in any way.

Finally, face recognition is context-specific in the sense that it makes sophisticated guesses: you pick up on similarities to the faces of others, and even an ambiguous or neutral facial expression will be interpreted as expressing an emotion. Type 1 processes function according to the principle that to a hammer anything is a nail; a process will selectively focus on certain aspects of the available information and rely on prior knowledge (Evans, 2008, p. 263). If some input is sufficiently similar to what the Type 1 process usually processes, it will run and interpret the input even if a different procedure would be more accurate (Kahneman, 2011, p. 97).

Many Type 1 processes are evolutionarily older.^3^ That means, their counterparts can also be found in other species. A nice example for this is our capacity to deal with small cardinalities. More concretely, many primates can “count” to three. They know for example that a bucket with three apples contains more apples than a bucket with two apples even if they cannot currently see them (Carey, 2009, p. 117). Human recognition of numbers smaller than three or sometimes four is also illustrative of Type 1 cognition. We recognise and manipulate such small cardinalities effortlessly and automatically.

Meanwhile, I described Type 2 cognition as slow, controlled, and rule-based. As an example take the activity of recalling everything that happened last Christmas. This is a process that has to be actively engaged. You may first try to recall where you were, by also thinking about where you were before Christmas, as well as how you travelled. Then you might think about who was there. You might invoke the rule that if George and Michael were there, then you also did sing because George and Michael always want to sing. Finally, you could recall what you did last Christmas. This is a slow, sequential process with many explicitly reasoned steps. Thus, reconstructing a coherent narrative of what happened last Christmas illustrates Type 2 cognition.

Type 2 cognition is sometimes argued to be unique to humans (Frankish, 2010, p. 922), and it underwrites many uniquely human capacities. For example, Type 2 cognition also underwrites our mathematical capacities for numbers greater than 3. Calculating how much flour, eggs, and sugar you need for a cake that is 1.66 times larger than in the recipe is another paradigmatic Type 2 activity. Applying the rule of proportion, you will need to go through several explicit steps to get to the desired results. Again this is a slow, controlled, and rule-guided process.

Although these two process types are distinct, they are part of the same cognitive apparatus. Sloman, for example, argues that while the two types operate differently, they are instantiated in the same ‘hardware’. They are also complementary: Type 1 tracks statistical features while Type 2 detects abstract properties (Sloman, 1996, p. 18).

This idea of complementarity of Types 1 and 2 is key. The two modes together enable adult human cognition. Type 1 cognition on its own would arguably be similar to an adult non-human great ape’s. Thus Type 1 enables basic access to and interaction with the world. Type 1 processes enable and constitute our perceptual faculties as well as basic agential abilities. Basic visual perception, for example, integrates a set of Type 1 processes that interpret the input from our visual nerves. Some of those processes use each other’s outputs; some run in parallel. The processes allowing movement detection and shape detection may for example run in parallel while the integration of our two eyes’ views into one stereo-picture uses already interpreted outputs of other processes.^4^

Type 2 cognition on its own is hard to even consider. Perceptual information would be raw and uninterpreted. With only Type 2 processes available, we would be confronted with a pure manifold, to use a Kantian term, of which we would hardly manage to make sense. Just already the noise generated by our sensory cells and nerves that our Type 1 processes normally filter out would be too much to handle. With only Type 2 cognition available, we would be cognitively helpless. Thus human cognition relies both on Type 1 and Type 2 processes which complement each other to enable our cognitive achievements.

A typical example for their complementarity is that Type 2 processes serve as a corrective to the ‘sloppy’ Type 1 processes (Evans & Stanovich, 2013, pp. 236–237). Given their context-specific workings, Type 1 processes sometimes produce mistaken beliefs that Type 2 processes can override. For example, the Muller–Lyer illusion is the product of visual Type 1 processes, and if you don’t know about it, you will form a mistaken belief about the lines’ relative length. However, your Type 2 capacities enable you to more closely examine the lines and correct the mistaken impression.

In general, Type 1 is much faster and less energy-consuming than the effortful and slow Type 2. The former can process vast amounts of raw information that would simply overload the latter. With active Type 2 cognition, we can *precisely* track three to four objects or properties at most while Type 1 processes can make *rough* estimates about much larger sets.

This efficiency, however, is also achieved through a high degree of specialisation, its context-specificity. Each Type 1 process can do *one specific thing*. Consider the specificity of facial recognition—people even discover Jesus’ face on toast. Type 2 compensates its comparative inefficiency with its universal applicability. From planning the construction of the Sagrada Familia or calculating the earth’s circumference to writing *Wuthering Heights*, it can (attempt to) solve almost any problem (Evans & Stanovich, 2013, p. 235; Stanovich, 2009, p. 22). Thus Type 2 cognition makes human cognisers independent from the particular contexts in which they evolved.

## Parallels

By now the *prima facie* parallels between dual-process theories and the virtue epistemologies should have become clear. Notably, there is the level of reflective access and control.

Type 1 processes run automatically, without any reflective access—we neither need to be aware of the process nor do we need to steer it. Given its commitment to externalism, reliabilist virtue also does not require reflective awareness or control. Consider for example our facial recognition abilities: they can be described both as automatically running capacities of Type 1 and, if we do not suffer from prosopagnosia, a virtue. We do not know how we recognise faces, we just do it.

Type 2 and responsibilist virtues on the other hand both essentially involve control and reflective access to what is going on. That is, Type 2 and responsibilist virtue manifest epistemic agency. Type 2 processes require (cognitive) effort to operate, as do responsibilist virtues. That is the role motivation plays for virtues: we can exert effort only if we are motivated to do so. Further, Type 2 processes involve explicit reasoning, while the responsibly virtuous agent must in principle be able to cite her reasons (Wright, 2010).

Additionally, both Type 1 and reliabilist cognition are often based on biological faculties that are innate and a product of evolution. Note however, that some Type 1 capacities and reliabilist virtues are acquired through training. Type 2 cognition and responsibilist virtues, in contrast, normally have to be acquired and developed through training or habituation.^5^ They rely on cultural practices; that is, both Type 2 activities and responsibilist virtues have to be transmitted between epistemic agents. For example, finding and executing a mathematical proof is a Type 2 activity—effortful, explicit, learned. A mathematically untrained person would not be able to do it. Proving a theorem also manifests responsibilist virtue—creativity, patience, diligence, which also have to be trained.

Finally, Stanovich (2009) argues that Type 2 consists of two distinct but interdependent systems. The first is the system that reasons explicitly and makes mental models; he calls this the ‘algorithmic mind’. The second controls and governs the former and involves ‘thinking dispositions’ like ‘conscientiousness, curiosity, diligence’ (Stanovich, 2009, p. 35) or ‘open-mindedness’ (Price et al., 2015). These thinking dispositions sound *just like* the responsibilist virtues.

All these parallels warrant exploring the view that the reliabilism-responsibilism distinction in virtue epistemology is grounded in the two process types that constitute human cognition. More specifically, I will argue that reliabilist virtues are dispositions of Type 1 cognition to function excellently and responsibilist virtues are dispositions of Type 2 cognition to function excellently (cf. Battaly, 2015).

I will call them Type 1 virtues and Type 2 virtues respectively. In the next two sections, I will give an account of how Type 1 and Type 2 virtues are constituted as well as what they can and cannot do.

### The reliabilist virtue of Type 1

Type 1 is in and of itself not guaranteed to be reliable. Note for example the already mentioned rationality wars which were triggered by Kahneman and Tversky’s (1973) studies on the dual-process account. Agents are highly unreliable in certain tasks when using Type 1 processes (Rysiew, 2008). This is partially due to the high degree of specialisation of Type 1 processes: their functioning is adapted to very specific contexts while outside of those contexts they become unreliable.

Take the example of our facial recognition module: it can discover Jesus’ face on a piece of toast. If left to its own devices, it recognises faces everywhere and generates the correspondent beliefs. Thus, other cognitive processes need to rein in the module’s output; if they don’t, we start seeing ghosts.

Reliabilist Type 1 virtue designates the excellent functioning of a Type 1 process. This does *not* mean that the process does what it was evolutionarily selected for; especially given the fact that we possess some Type 1 capacities that are learned, e.g. reading. Epistemic reliability and evolutionary success may come apart (Churchland, 1987, pp. 548–549).

Rather, Type 1 virtue indicates the epistemic reliability of a cognitive process: the tendency to produce many true beliefs and avoid false ones. I resist naturalising the normative side of reliabilist virtues. There may be Type 1 processes that are epistemically virtuous, because highly reliable, while they suboptimally contribute to a species’ propagation. Vice versa there may be evolutionarily highly adaptive traits that are epistemically vicious. Rabbits, for example, are highly sensitive to predators, but they often run away from mere shadows. This is not epistemically virtuous, but it secures the rabbits’ survival.

I restrict the normative domain of Type 1 virtues, their excellency, to their reliability. Arguably, that is what Type 1 processes do best epistemically. Type 1 processes are the elementary tools that we need to get basic epistemic access to our environment. For that access we need reliability. Additionally, I do not see what other sort of epistemic value Type 1 processes might systematically deliver given their simple input–output structure. Type 1’s reliabilism is also suggested by the fact that Type 1 processes usually are evolutionarily older; cognising organisms need some reliable cognitive processes.

Please note that this account is somewhat revisionary about reliabilist virtue. First, Type 1 virtues are much more fine-grained than typical reliabilist faculties. Type 1 virtues are excellences of single cognitive modules or algorithms that make up single Type 1 processes, rather than the more complex processes that make up entire faculties like visual perception. This helps to clearly individuate Type 1 virtues by simply following the individuations that cognitive psychologists make.

Second, on classical reliabilist accounts, the very faculties are the virtues: eyesight, memory, hearing. I suggest instead, that the excellent functioning of these faculties, i.e. their *disposition* to operate reliably, is their virtue. This transforms reliabilist virtues from faculties into dispositional properties of faculties, which is in keeping with more recent agent reliabilist accounts (Sosa, 2015, p. 44).

This move serves two purposes: one, it permits assimilating reliabilist and responsibilist virtues which are also dispositional. Two, it distinguishes clearly between processes and their virtue: most individuals arguably possess the same process types, but they are not all equally virtuous. I prefer variance in epistemic performance to be accounted for by variance in agents’ processes’ reliability—that is variance in their excellency—rather than by variance in what type of process the agents have at their disposition. My myopic eyesight is fairly unreliable for distant objects, but it is constituted by the same *process types* as my sharp-sighted counterpart’s. We differ in the processes’ properties, notably the range of parameters at which they are reliable, as I am only reliable with nearby objects.

### Responsibilist virtue of Type 2

If we continue in this track, then a responsibilist Type 2 virtue is a disposition of Type 2 processes to function excellently. As with Type 1, Type 2 processes are not guaranteed to function perfectly, there may be hosts of mistakes. But, because processes of Type 2 are domain-general which means that they can be used to solve any problem independently of context (Evans, 2008, p. 261), their mistakes are of a different kind than those of Type 1 processes. Mistakes of Type 2 processes are failures due to inattention, lack of capacity, laziness, or something of the sort rather than due to operating in an inappropriate context.

Type 2 does not and cannot provide the subject with basic epistemic access to the environment. It is not usually used to produce a representation of the organism’s immediate environment, as it would be much too slow for that. Therefore, although we may expect it to reliably deliver truths in some tasks, e.g. mathematical proof, it is not necessarily subject to the same constraints of reliability as is Type 1.

Instead, Type 2 cognition is an extremely versatile tool that can be used for universal problem solving. These problems reach from how to seat guests at a wedding to developing an algorithm that extrapolates a picture of a black hole. Stanovich (2009, p. 28) argues that many of these Type 2 capacities rely on the ability to ‘decouple’, that is to have representations without endorsing them as accurate. Thus Type 2 may also be used to develop the counterfactual scenario of what would happen if kangaroos lacked tails. Successful Type 2-activity then does not always aim at accuracy.

Solving a problem, say finding out whodunnit, can itself be split into independent subprocesses. First, one needs to ask the right questions to match the problem. When did the crime occur? Who was there? What are possible motives? Second, one needs to generate relevant possible avenues for responses. For example, that the niece did it for the inheritance or the gardener because he’s the gardener. Third, one needs to run through the different alternatives to pick one. Say, that most likely the niece did it given her motive. Fourth, one needs to test the favoured response against defeaters, e.g. the niece’s purported alibi.^6^ Each of these subtasks has different success conditions, reliability only applies straightforwardly to the third step. The diverse operations that Type 2 cognition executes—inferential reasoning, mental modelling, etc.—all contribute to solving problems without thereby already reliably delivering truth.

A Type 2 virtue is then a disposition of Type 2 cognition to execute such tasks that are related to solving a problem or answering a question (Sosa, 2015, p. 53) well. There is for example the virtue of open-mindedness which is the disposition to consider alternatives that go against one’s prior beliefs. Perseverance generally supports problem solving; it is the virtue of sustaining one’s effort when solving a problem. Imaginativeness is the capacity to virtuously manage one’s representations, the ability to develop relevant alternatives, compare them, and keep track of details.

Responsibilist virtue is usually taken to require a motivational part. In order to be intellectually virtuous, agents need to care about the truth (Montmarquet, 1992). How does that relate to Type 2 virtues? As mentioned, Type 2 processes require cognitive effort. Necessarily, we need to be motivated in order to expend effort, especially across an extended process. I would argue that the most excellent epistemic performance of Type 2 processes across the board will require a desire for truth. Other motivations may well yield good epistemic results, but they can easily side-track the process and produce some other value than truth or understanding as soon as it becomes pragmatically opportune. I therefore take a motivation for truth to be necessary for virtue of Type 2 processes.

Note also that the problem-solving ability of Type 2 and its virtue is a way of generating knowledge: knowledge how to solve problems. It does this constitutively, not only in an auxiliary role as the agent reliabilist would have it. On the agent reliabilist picture (Sosa, 2015, p. 42) the responsibilist virtues work to make the necessary information accessible to the reliabilist virtues. Responsibilist virtues “put you in a position to know” but cannot constitute knowledge (Sosa, 2017, p. 144).

On my view however, Type 2 virtues do more: they produce the relevant beliefs. While the Type 2 virtue may draw on input from Type 1 faculties, it will process them and produce the relevant knowledge independently of the operation of Type 1 processes. I do rely on eyesight to read my calculations on a sheet of paper, but the process producing the belief in the result is Type 2. In this case Type 1 virtues are auxiliary to Type 2 virtues. Without the Type 2 process we would be incapable to even grasp the process’s output. Thus the Type 2 virtue is constitutive of the knowledge how to solve a problem.

One difference to Type 1 is that Type 2 is not operating and generating beliefs by default. Instead it is only activated when required. The disposition to use Type 2 cognition only when it is actually required is itself an epistemic virtue. According to Stanovich, thinking dispositions or cognitive styles govern when algorithmic processes are called upon (Stanovich, 2009, p. 38). For example, overuse of algorithmic Type 2 processes leads to the Type 2 vices of rumination and indecisiveness while underuse produces the vicious habit of jumping to conclusions.

The explicit processing of Type 2 contributes to another particularity of Type 2 virtue. To transmit knowledge, we need to formulate it explicitly. That is, sharing knowledge by language frequently needs to go through Type 2 processing. This is also necessary to express testimony clearly, concisely, and convincingly.

Additionally, testimony is generally open to legitimate requests for justification. The practice of justifying will also involve processes of Type 2: the invocation of reasons (cf. Lepock, 2011, p. 120) will fall to Type 2 virtues even if that knowledge originally was a product of Type 1. Responsibilist virtue then also plays a key role in the transmission of knowledge: to be a reliable testifier you usually need to be responsible.

We may wonder whether Type 2 virtues are really acquired by habituation, i.e. learned: yes and no. A good short-term memory supports the excellent functioning of Type 2 cognition because one can hold more considerations in one’s mind. Is it acquired through habituation? Not necessarily; while it can be trained, it is different from classic responsibilist virtues like epistemic humility that do not seem to presuppose sheer brainpower. In a way, this makes some Type 2 virtues more similar to Aristotle’s (2004, p. 25) analogy of virtue with strength, which is also partially innate but needs to be trained to become fully virtuous.

Still, many of the Type 2 virtues that lead to an excellent functioning of Type 2 must be learned. It is one of the reasons why aspiring philosophers are taught formal logic: it instils them with dispositions to interact with epistemic problems in a virtuous manner. Baby-logic nurses the epistemic virtues.

Some authors take perfect responsibilist virtue to be *automatic*. This is incompatible with Type 2 processes essentially requiring effort and reflexion. I want to argue that something else happens in this case: if a virtue has been perfected, the Type 2 habit has become so deeply ingrained that it becomes a Type 1 process. That means, the Type 2 virtue has become so effortless and automatic for this agent that it now is a Type 1 virtue.

Indeed, dual-process theories allow that a Type 2 process may become so automatic that it becomes a Type 1 process (Kahneman & Frederick, 2002, p. 51). That is, a virtue may, depending on the agent, either be a Type 1 virtue or a Type 2 virtue. Consider for example recognitional abilities: while you may initially have to effortfully reason yourself to the conclusion that this is a linden tree, with time your recognitional abilities become so automated that they shift into Type 1 cognition and they become part of a Type 1 virtue.^7^ Another example is reading: for new readers, reading is an effortful Type 2 activity. Every syllable has to be spelled out and composed into a word; these words have then to be synthesised into a sentence. Experienced readers on the other hand may be able to grasp entire sentences automatically and at once. Thus, the ability to read can either be a Type 2 or a Type 1 virtue.

This account of responsibilist virtue as excellence of Type 2 cognition is also revisionary about responsibilism. Instead of habits of an agent, Type 2-virtues are dispositions of our cognitive system. Motivation is causally but not constitutively necessary for responsibilist virtue; in this sense it is closer to agent reliabilism. Nevertheless, there is a morally loaded notion of wisdom as fully developed Type 2 virtue: desire for truth will steer an agent to learn everything about something, *including* the pertinent moral truths (Plato, 2005, pp. 88b–89a). Oppenheimer, for example, was only partially virtuous when he developed the a-bomb. While the development of such a device in the face of many technical difficulties manifested partial Type 2 virtue, it included a failure to consider its moral implications.

## Solving the generality problem and the situationist challenge

In the preceding section, I developed an account of epistemic virtue that is underwritten by dual-process theory. I will now show that, beyond this naturalisation, my approach can also deal with two of the most prominent challenges to virtue epistemological accounts. First, this is the venerable *generality problem* (Conee & Feldman, 1998); second there is the *situationist challenge* (Alfano, 2013).

The generality problem asks how to individuate the cognitive processes that are evaluated for their reliability in a principled and epistemologically plausible manner. (Conee & Feldman, 1998, pp. 3–5) Let’s take Conee’s and Feldman’s example of recognising maple trees: what factors are part of my virtue to recognise tree species? I could for example gerrymander a virtue such that, always when I am near maples, I reliably recognise maples; unfortunately, in other contexts I also mistake plenty of other trees for maples because I simply call all trees with big leaves maples. Which of the two descriptions of the capacity is to be evaluated for its reliability and why? My reliably recognising maples when near maples or my unreliably mistaking many trees for maples?

Part of cognitive psychology’s research project is to individuate the different specialised process types that make up cognition, e.g. the facial recognition module. This project is completely independent from virtue epistemology; it is therefore a strongly principled solution to the generality problem. I think it also is epistemologically plausible. This psychological approach has already been proposed by Goldman (1979, pp. 96–97) and seen different specifications by Alston (1995, pp. 12–13), Beebe (2004) and Kampa (2018), as well as by Lyons (2019).

My approach can be fruitfully compared to Lyons’s (2019) algorithms and parameters solution to the generality problem. Lyons’s algorithms are the psychological capacities whose excellence constitutes Type 1 virtues on my view. Because his project is to explain which cognitive processes generate justification, individuating by algorithms is too coarse for Lyons. He instead evaluates algorithm-parameter pairs for reliability.

Parameters are factors that are neither part of the algorithm’s input nor of the algorithm itself but which nevertheless influence its output as a matter of psychological laws. That is, parameters are extraneous influences on the algorithm that may influence its reliability. In order to avoid the trivialization of external influences—e.g. “when standing near maple trees”—only factors that influence the algorithm’s functioning according to a psychological law that governs the algorithm count as parameters. For example, background noise is a parameter because, as a matter of psychological law, we can extract less information over background noise. An algorithm paired with its parameters individuates a process that is evaluated for reliability (Lyons, 2019, pp. 473–474).

Interestingly enough, the parameters also play a role for Type 1 virtues: the stronger an algorithm is, i.e. the less that variation in parameters impinges on an algorithm’s reliability, the more the algorithm is virtuous. Type 1 processes usually are highly context-specific, they only work reliably within a narrow range of parameters. To be properly virtuous, a process needs to be reliable across as broad a range of contexts or parameters as possible. Call the range of contexts in which the process runs reliably its strength. A Type 1 virtue is a disposition of a Type 1 algorithm to be strong, i.e. to remain reliable across wide ranges of parameter values.

Another way for a Type 1 algorithm to remain reliable and virtuous is that it can have high trigger reliability. For that, it must be sensitive to its context; a trigger reliable algorithm avoids running at parameters where it is unreliable (Alfano, 2013, p. 151). In sum, the generality problem is solved by appealing to the process individuation of cognitive psychology. Meanwhile, my account does not need to individuate algorithm-parameter pairs. Instead, a virtue’s excellence is evaluated by examining the reliability of a single algorithm across its entire range of parameters.

The dual-process account of virtue can also deal with the situationist challenge. Situationism argues that human cognition is highly sensitive to variation in contexts or parameters which undermines the notion of virtues as *stable* excellent dispositions (Alfano, 2013, p. 120). Both Type 1 and Type 2 capacities can be strongly influenced by epistemically irrelevant factors, e.g. your blood sugar.

The account of Type 1 virtues as being stable across parameters, i.e. strong and trigger reliable, responds to exactly this challenge. Situationists may discover epistemically irrelevant factors; however, these factors influence the capacity as a matter of psychological law. Consequently, being stable across situations is part and parcel to Type 1 virtue. If someone’s Type 1 capacity breaks down at certain parameters, e.g. low blood sugar, then it is simply a little less virtuous because it is less strong. However, this can be compensated through a raised trigger reliability, notably through Type 2 monitoring where judgment may be suspended because of the low blood sugar.

This brings us to Type 2 virtues. Situationists also argue that our Type 2 cognition is influenced by extraneous factors to a degree that undermines the notion of stable cognitive dispositions, i.e. virtues. However, cognitive styles are argued to be a stable phenomenon (Kozhevnikov, 2007); thus, cognitive psychology itself argues for a stable foundation for virtues, and cognitive psychologists are not unanimous about the nature of cognitive dispositions.

Additionally, we can co-opt the same strategy as we did for the generality problem: the monitoring and steering processes that arguably constitute a cognitive style (Kozhevnikov, 2007; Zhang & Sternberg, 2005) are more virtuous if they support problem solving across a greater variance in parameters. To return to our previous example, the situationist argues that our logical reasoning capacity is much worse if we are hungry, and a disposition that disappears once we are hungry is not a real virtue. Given the governing and monitoring function of Type 2 virtues, we can argue that in a virtuous agent the monitoring Type 2 virtues would suspend Type 2 activity until she has eaten something and her cognition is again functional. In a way, the situationist challenge is incorporated into the concept of epistemic virtue.

## Complementary virtues

Type 1 and 2 cognition are unified because they are part of a single organism’s cognitive apparatus. Both are key to explaining human cognition as it really occurs. In this apparatus, they play complementary roles.

If reliabilist virtue is excellence of Type 1 processes and responsibilist virtue is excellence of Type 2 cognition, then these epistemic virtues are also complementary. Note, that this unification is not teleological: the virtues are not unified because they directly or indirectly aim at the same monist norm, e.g. truth or reliability. My account is no thematic unitarianism (Goldman, 2002).

Instead, my account is parasitic upon cognitive psychology’s unifying explanations. As a presumably successful explanation of human cognition, dual-process theory unifies the two types of cognition. My theory develops epistemologically distinct accounts of virtue that are however indirectly unified through their cognitive foundations. Additionally, my account draws on the cognitive complementarity of Types 1 and 2 to argue for the epistemic complementarity of their epistemic virtues.

Consider how Battaly’s (2015) Pluralism fares on this count. There, the shared foundation is the agent in the abstract which involves a reliabilist cognising organism but also a responsibilist epistemic agent. It is too weak to simply claim that both reliabilist virtues and responsibilist virtues are epistemic excellences; plenty of other qualities might be introduced as virtues otherwise, e.g. your ears or even your hands because you may use them to dig up things and learn what is underground. Additionally, a unified account here would have to go to the very foundations of epistemic activity, explaining why both reliabilist and responsibilist qualities are epistemic excellences; it is not obvious that this can be done. Recall the unity challenge for pluralism (Pedersen, 2017). My account simply naturalises the distinction and explains that this is what our cognitive capacities do best while pointing to distinctions from human cognitive psychology, i.e. dual-process theory.^8^

Consequently, reliabilist and responsibilist virtues are on the one hand unified through their grounds. On the other hand, they are unified in their functional complementarity: neither responsibilism nor reliabilism on its own is able to account for the full range of successful human cognition. The two virtue-types describe the good functioning of two distinct *modi operandi* of the human cognitive system (cf. Fleisher, 2017).

I believe that reliabilism and responsibilism respectively focused on the essential functioning of one of our types of cognition, overlooking that the functions of both types are necessary to account for human epistemic success. This is how the split in virtue epistemology came about.

Virtue reliabilism is based on the recognition that the naked operation of Type 2 processes, as they are manifested in Cartesian epistemology, is utterly sterile and doomed to never get off the ground because it lacks an efficient interface between the organism and the environment. Meanwhile, virtue responsibilism recognises the limits of merely having reliable cognition which would never permit certain cognitive achievements that go beyond just reliably true belief. Thus, contemporary epistemology cannot do without accounting for the full range of capacities of Type 1 and Type 2 cognition and thereby both reliabilist and responsibilist virtue.

Finally, this gives us an account how we can naturalise Aristotelian virtues. Standard responsibilist accounts use the fairly vague and hard-to-pin-down terminology of disposition and habit *of an agent*. Basically, anything can be a habit or a disposition of an agent. There seem to be few to no restrictions as to what sort of modal property might fall into this category. This leaves a large explanatory gap in the account that, I believe, motivated the situationist challenge (Alfano, 2013; Doris, 2002).

If we tie responsibilist virtues to Type 2 and reliabilist virtues to Type 1, then there are clear requirements on what has to go on with the agent if a disposition is to be a virtue. Namely, that a Type 1 or 2 process has to occur in appropriate contexts and to operate successfully. Essentially, we are able to tell what sort of property an epistemic virtue is and what role it plays for us as cognising organisms.

## Competing accounts of epistemic virtue

Dual-process theories are best known from the heuristics and biases debate (Kahneman, 2011). This debate mostly focuses on the deficiencies of these cognitive systems and operates with a restrictive notion of rationality such as Bayesianism or logical coherence rather than an organism’s broader epistemic success. This focus on defects of the two types has distracted from what it means for them to function successfully.

The focus on deficiencies of dual-process cognition is also visible in some virtue epistemology papers that explicitly deal with dual-process theories. Most notably, Church and Samuelson (2015) argue that an *imbalance* between Type 1 and Type 2 processes leads to the responsibilist vice of intellectual arrogance. The corresponding virtue of intellectual humility is a product of Type 2 correcting deficient Type 1 processes.^9^

I agree that the Type 2 virtue of humility may correct deficient functioning of Type 1 by improving its trigger reliability, but this omits Type 1 processes’ potential to function reliably and thereby virtuously on their own. Intellectual humility was only required in these cases because our Type 1 cognition was already deficient: had the processes not been executed, that is had the disposition been trigger-reliable, then the process would not have been vicious. Rather, it would have been virtuous by operating only in appropriate contexts. Additionally, intellectual arrogance arguably does not only designate an overreliance on Type 1 but also an overconfidence in one’s Type 2 cognition.

Meanwhile, Mi and Ryan (2020) argue that reliabilism and responsibilism are unified through our dual-process cognition. They suggest that Type 1 and Type 2 cognition both underwrite the responsibilist “master virtue of skilful reflection” which guides and steers both our reliabilist and responsibilist virtues. Thus their account does not specify the particular roles of Type 1 and Type 2 cognition respectively but unifies the virtues through the guidance of their master virtue. I would however argue that Type 1 virtues may function very well without being nannied around by a responsibilist master virtue. Thus, Mi and Ryan’s (2020) account is the responsibilist counterpart to Lepock’s (2011) reliabilist annexation project.

Axtell (2017) limits the workings of dual processes to reliabilist virtue. In defence against the situationist challenge, he argues that both virtues of Type 1 and Type 2 are reliabilist. Indeed, in general you might wonder why we should not simply subsume all reliable processes of our cognitive apparatus under the banner of reliabilist virtue and ignore responsibilist virtue entirely.

I believe that limiting Type 2 virtues to the constraints of reliabilist virtue does not account for the full range of their capacities. Reliability may account for part of its epistemic success, but there are Type 2 capacities whose successes are not reducible to bare reliability.

As a first point in case, consider the thinking dispositions that Stanovich (2009) proposed as a part of Type 2: these cognitive styles simply do not look anything like reliabilist virtues (Lyons, 2019, p. 485). The reliabilist would start to pitch her tent so wide as to simply include responsibilist virtues undigested in her theory, something that Axtell (2017, p. 18) does. By undigested, I mean that such a reliabilist theory takes a fully developed responsibilist thinking disposition but only considers its reliability to constitute its virtue.^10^ This then fails to account for other epistemic values like understanding that virtuous thinking dispositions also produce, and it will exclude some apparently virtuous thinking dispositions from being virtues because they do not raise reliability. This is an annexation project and it artificially separates reliable thinking dispositions from other thinking dispositions that make you a better cogniser.

The reliabilist might instead concede that cognitive styles are the foundation for responsibilist virtues but insist that the algorithmic Type 2 cognition is reliabilist. After all, Stanovich (2009) talks of a tripartite model of the mind. This would be a minimalist version of my view.

However, responsibilist virtues are better suited to account for the excellent functioning of Type 2 cognition in general. Type 1 processes really appear to have one single epistemic function: bare-naked reliability. Meanwhile, the capacities that make up Type 2 produce a much broader range of epistemic values than just true beliefs, just like responsibilist virtues do.

While some of these capacities may be able to reliably generate true beliefs, others would at best, if at all, be indirectly reliable. Many processes of decoupled thinking, e.g. the imagination, do not usually generate true beliefs at all. When Mary Shelley invented Frankenstein and his monster, it was definitely an intellectually virtuous performance but hardly reliable. The same goes for dispositions like open-mindedness. These Type 2 dispositions may nevertheless be epistemically valuable and thereby virtues.

Finally, the algorithmic mind is generalist, it can apply its capacities to *anything*. Its processes are not as individuated and context-specific as those of Type 1 thus making the generality problem even more virulent for evaluating the reliability of Type 2 processes than for Type 1 processes. In order to function reliably, the algorithmic mind needs to be governed by excellent thinking dispositions, i.e. responsibilist virtues.

Stanovich illustrates this with the example of G.W. Bush who apparently had a powerful algorithmic mind with an estimated IQ of 120. At the same time he was narrow-minded and lacked in curiosity (Stanovich, 2009, pp. 1–2). If the algorithmic mind were reliabilist, then Bush would have to be an excellent cogniser; however, he is well-known for his intellectual shortcomings. In other words, without responsibilist virtue, Type 2 cognition cannot function well or even reliably (Stanovich, 2009, p. 43).

If we are to take the idea that epistemic virtues are grounded in our cognitive apparatus seriously, then I believe that making the cut between reliabilism and responsibilism along the line between Type 1 and Type 2 is the most natural. It is an elegant way to solidly anchor virtue theory in our cognitive architecture.

## Footnotes

1 More recently Miracchi (2015) and Kelp (2018) have argued for a knowledge-first virtue reliabilism.

2 I would like to thank an anonymous referee for pointing out this connection.

3 However, there are also younger Type 1 capacities, notably many elementary human linguistic abilities are of Type 1. Additionally, some Type 1 capacities are learned—consider for example the ability to read.

4 This is not a complete account of visual perception; it simply is supposed to show that it consists of many Type 1 processes, including facial recognition.

5 Some agents manifest innate responsibilist virtues, e.g. mathematical knowledge in prodigies, however this clearly is the exception. This points to an additional important trait of both Type 2 and responsibilist virtue: their foundations are already present in most agents and in some agents they may develop spontaneously into virtues. Below, I further examine this feature, comparing virtues of Type 2 with strength which also has innate foundations.

6 This is merely a sketch of what problem solving requires in the abstract. Some problems may require more or less steps. For a highly developed account of virtue and problem solving see Morton (2012).

7 Millar (2009) individuates recognitional abilities teleologically by whether they de facto produce knowledge. Meanwhile, I advocate individuating them through the cognitive processes subserving the ability. These dispositions may then be more or less knowledge-conducive.

8 Consequently, my account of epistemic virtue is anthropocentric or centred on organisms with a brain while Battaly’s theory aims to be more universalist. However, her account does not contain a theory of what e.g. a robot’s cognitive excellence would be either, and it is not obvious to me that a robot needs both responsibilist and reliabilist virtues. It might need an entirely different kind of epistemic virtue.

9 A similar idea can be found in Miranda Fricker’s *Epistemic Injustice* (2007), where she argues that the virtue of epistemic justice corrects for our racist implicit (Type 1) biases.

10 This is particularly remarkable because Axtell does not reject responsibilist virtues; he just doesn’t associate them with thinking dispositions.
